# Supplementary material for: Development of a Guideline to Enhance the Reporting of Concept Mapping Research: Study Protocol
Source: Int J Environ Res Public Health. 2022 Jun 14;19(12):7273. doi: 10.3390/ijerph19127273 (PMC9223457; doi:10.3390/ijerph19127273)
Supplement: Supplementary file 1 [file ijerph-19-07273-s001.zip › Table S2_MEDLINE Search strategy.pdf]

Medline Search

S. No	Searches
1	Concept mapping study.mp.
2	concept map*.mp.
3	concept mapping.mp.
4	structured conceptualization.mp.
5	1 or 2 or 3 or 4
6	Ariadne.mp.
7	concept systems.mp.
8	6 or 7
9	5 or 8
